# Anti-Schistosomal Activity of Cinnamic Acid Esters: Eugenyl and Thymyl Cinnamate Induce Cytoplasmic Vacuoles and Death in Schistosomula of *Schistosoma mansoni*

**DOI:** 10.3390/molecules200610873

**Published:** 2015-06-12

**Authors:** Jan Glaser, Uta Schurigt, Brian M. Suzuki, Conor R. Caffrey, Ulrike Holzgrabe

**Affiliations:** 1 Institute of Pharmacy and Food Chemistry, University of Wuerzburg, Am Hubland, D-97074 Wuerzburg, Germany; E-Mails: jan.glaser@uni-wuerzburg.de (J.G.); u.holzgrabe@pharmazie.uni-wuerzburg.de (U.H.); 2 Institute for Molecular Infection Biology, University of Wuerzburg, Josef-Schneider-Str. 2/D15, D-97080 Wuerzburg, Germany; 3 Center for Discovery and Innovation in Parasitic Diseases, University of California, San Francisco, 1700 4th St., San Francisco, CA 94158, USA; E-Mails: brian.suzuki@ucsf.edu (B.M.S.); conor.caffrey@ucsf.edu (C.R.C.); 4 Department of Pathology, University of California, San Francisco, CA 94158, USA

**Keywords:** *Schistosoma*, schistosomula, parasite, eugenyl cinnamate, thymyl cinnamate, vacuoles, autophagy, anti-schistosomal activity

## Abstract

Bornyl caffeate (**1**) was previously isolated by us from *Valeriana (V.) wallichii* rhizomes and identified as an anti-leishmanial substance. Here, we screened a small compound library of synthesized derivatives **1**–**30** for activity against schistosomula of *Schistosoma (S.) mansoni*. Compound **1** did not show any anti-schistosomal activity. However, strong phenotypic changes, including the formation of vacuoles, degeneration and death were observed after *in vitro* treatment with compounds **23** (thymyl cinnamate) and **27** (eugenyl cinnamate). Electron microscopy analysis of the induced vacuoles in the dying parasites suggests that **23** and **27** interfere with autophagy.

## 1. Introduction

Schistosomiasis, also known as bilharzia after its discoverer Theodor Bilharz [1], is one of the most common chronic diseases of poverty. The disease is found in sub-Saharan Africa, parts of Asia and South America [2,3], and is caused by trematodes of the genus *Schistosoma* which can survive in the human host for years or even decades [4]. Of the three principal species infecting humans, *S. mansoni* is the most prevalent [5] causing intestinal schistosomiasis. Infection occurs during contact with freshwater bodies that contain the appropriate species of infected snails (*Biomphalaria* spp. [6]). Free-swimming larvae (*cercariae*) penetrate the skin and reach the bloodstream where they develop into adult worms. The infection exhibits a mortality rate of approximately 300,000 deaths a year [7] but is more commonly chronic and morbidly debilitating. Disease caused by *S. mansoni* infection is associated with chronic hepatic and intestinal fibrosis, internal varices, which, if located close to the stomach or esophagus can result in hematemesis [8,9]. With *S. haematobium* infections, a variety of urinary system complications can result [10].

In 2012, 249 million people worldwide were in need of preventive treatment for schistosomiasis with praziquantel (PZQ), the only drug available that is active against all species of *Schistosoma* [11,12]. Although there are no reports of clinically relevant resistance to PZQ, recent commitments to increase access to the drug mean that we should remain vigilant in the event resistance does arise [13]. It is, therefore, advisable to look for alternate drugs to fight this disease.

We recently discovered that bornyl caffeate (**1**) from the rhizomes of *Valeriana (V.) wallichii* possessed anti-leishmanial activity [14]. We followed up on this initial finding with the synthesis of a small compound library of 28 systematically varied derivatives [15]. Here, we screened the entire library (except the cytotoxic acetylated bornyl caffeate) plus three additional compounds (**18**, **28** and **29**) for *in vitro* activity against *S. mansoni* schistosomula (post-infective larvae) and compounds **23**, **27**–**30** for *in vitro* activity against adult worms.

## 2. Results and Discussion

The compound library and the test results are summarized in Table 1 and Table 2. Screens were performed at 5 μM and 10 µM against schistosomula and adults, respectively, over the course of 3 days. A previous constrained nomenclature is used to record phenotypes [16] (every 24 h) that describe both motion and morphological changes. For schistosomula, the terms employed are ‘dead’ (D), ‘degenerate’ (Deg), ‘round’ (R), ‘overactive’ (O) and ‘dark’ (Dark). After incubation with **23** and **27**, we also noted the development of internal ‘vacuoles’ (V), which presaged the eventual death of the parasite.

### 2.1. Screen Results

The bornyl caffeate **1** originally isolated and identified from *V. wallichii* was inactive, whereas some of its derivatives proved to be far more potent. In any case, compound **1** shows typical PAINS [17] characteristics, namely the catechol moiety and the α,β-unsaturated double bond that are known to give false positive results in multiple biochemical assays. As the catechol moiety is prone to oxidation that results in the formation of an *o*-benzoquinone [18], catechols can act as antioxidants or pro-oxidants, which can induce damage to DNA or proteins and therefore may result in antiparasitic activity. Omitting the vicinal phenolic hydroxyl groups improved anti-schistosomal activity and reduced cytotoxicity significantly. This was the case with bornyl cinnamate (**3**) as well as thymyl (**23**) and menthyl cinnamate (**26**), which produced stronger phenotypes than their respective catechol counterparts (**1**, **21** and **24**; see Table 1 and Table 2).

**Table 1:** Results of the screening of compounds **1**–**10** on schistosomula (Phenotypes: D = dead, Deg = degenerate, O = overactive, R = round; severe phenotypes are in bold). PZQ = Praziquantel.

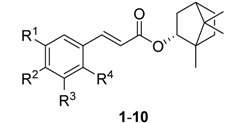					Observed Phenotype After (c = 5 μM)			
Compound	R^1^	R^2^	R^3^	R^4^	24 h	48 h	72 h	J774.1 [μM] [15]
**1**	-OH	-OH	-H	-H	-	-	-	8.3
**2**	-OH	-OCH_3_	-H	-H	R	R, Dark	R, Dark	48.7
**3**	-H	-H	-H	-H	-	R, Dark	R, **Deg**	45.2
**4**	-H	-H	-Cl	-Cl	R	-	-	58.9
**5**	-H	-Cl	-H	-Cl	R	-	-	60.6
**6**	-H	-OCH_3_	-H	-H	R	-	-	42.6
**7**	-H	-Br	-H	-H	R	-	Dark	54.6
**8**	-H	-N(CH_3_)_2_	-H	-H	R	-	-	>100
**9**	-H	-OBn	-H	-H	R	-	-	>100
**10**	-H	-Cl	-H	-H	-	Dark	-	49.5
PZQ	-	-	-	-	O, Dark	O, **Deg**	**Deg**	-

The α,β-unsaturated double bond represents an potentially reactive Michael system. However, only the bornyl (**3**), menthyl (**25**, **26**), thymyl (**23**) and eugenyl cinnamates (**27**) induced the severest phenotypes. Accordingly, the existence of a Michael system does not necessarily lead to anti-schistosomal activity. Also the IC_50_ of the J774.1 cell line seemed not to be decreased by this Michael system.

The data also show that cytotoxicity appears not to correlate with anti-schistosomal activity. Compounds with highest cytotoxicity (**1**, **11**, **21**, **24**) have the lowest effect on schistosomula. The most potent anti-schistosomal activity is achieved by maintaining the aromatic ring of the cinnamoyl in the absence of substituents.

Overall, the eugenyl cinnamate **27** is the most potent schistosomicidal compound. By 24 h, the compound had caused degeneration of the schistosomula with the formation of vacuoles. After 72 h all the parasites were dead. Similarly, the thymol cinnamate **23** also led to the formation of vacuoles (V) and degeneration (see Table 2).

**Table 2:** Results of the screening of compounds **11**–**30** on schistosomula (Phenotypes: D = dead, Deg = degenerate, O = overactive, R = round, V = vacuoles; severe phenotypes are in bold). PZQ = Praziquantel.

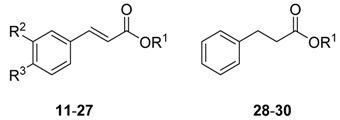				Observed Phenotype After (c = 5 μM)			
Compound	R^1^	R^2^	R^3^	24 h	48 h	72 h	J774.1 [μM] [15]
**11**	isoborneol	-OH	-OH	-	-	-	8.8
**12**	isoborneol	-OH	-OCH_3_	-	R	-	44.3
**13**	isoborneol	-H	-H	-	-	-	46.0
**14**	isoborneol	-H	-NO_2_	-	-	-	>100
**15**	cyclohexanol	-H	-H	-	-	-	>100
**16**	geraniol	-H	-H	-	-	-	>100
**17**	adamantol	-H	-H	-	-	-	46.7
**18**	4-thujanol	-H	-H	-	-	-	>100
**19**	naphthol	-H	-H	-	-	-	44.5
**20**	α-bisabolol	-H	-H	R	R	-	>100
**21**	thymol	-OH	-OH	O	-	-	8.8
**22**	thymol	-OH	-OCH_3_	-	R, Dark	Dark, R	45.6
**23**	thymol	-H	-H	**Deg, V**	**Deg, V**	**Deg**	44.8
**24**	menthol	-OH	-OH	-	R, Dark	Dark, R	2.0
**25**	menthol	-OH	-OCH_3_	R	R, Dark	R, **Deg**	45.6
**26**	menthol	-H	-H	R	R, Dark	R, **Deg**	44.8
**27**	eugenol	-H	-H	**Deg, V**	**Deg**	**D**	32.2
**28**	thymol	-	-	-	-	-	>100
**29**	eugenol	-	-	-	-	-	>100
**30**	borneol	-	-	-	-	-	>100
PZQ	-	-	-	O, R	O, **Deg**	**Deg**	-

The most active cinnamates were again synthesized without the double bond (**28**–**30**). However, these were inactive. By comparison, the bornyl phenylpropionate (**30**) retained anti-leishmanial activity [15]. For schistosomula, it is possible that the Michael system is responsible for inducing the vacoules—as seen with **23** and **27**—and is an essential component to the pharmacophore.

Finally, **23** and **27**–**30** were selected for screening against adult worms. No effect on the parasites was observed at a concentration of 10 µM over the course of 3 days (data not shown). The vulnerability of the schistosomula may be due to the major transcriptional, translational and structural changes taking place as part of the adaptive process to the mammalian host, in contrast to adult worms which are fully adapted.

### 2.2. Analysis of Vacuoles

**Figure 1:**
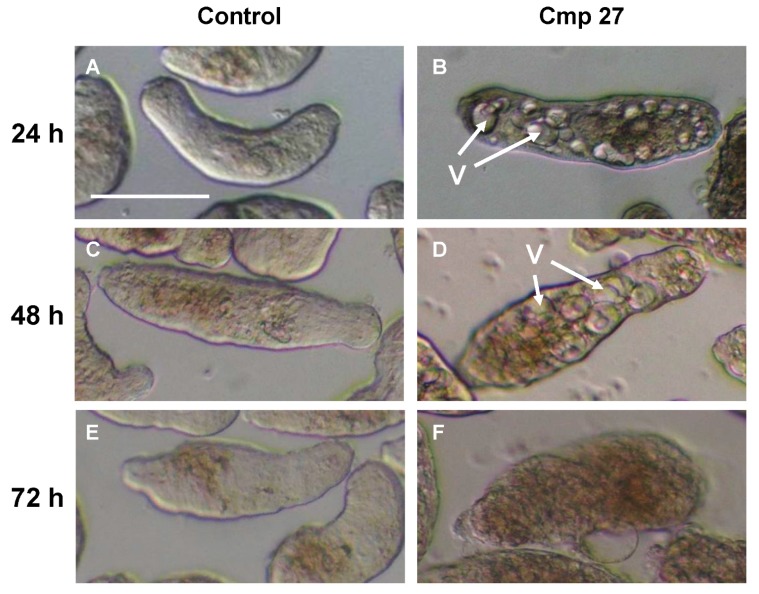
Microscopic images of schistosomula; control group (**A**,**C**,**E**) and treated with 5 µM **27** (**B**,**D**,**F**) after 24, 48 and 72 h. The vacuoles (V) are prominent after 24 and 48 h, whereas by 72 h the somules are dead. Scale bar = 0.2 mm.

Vacuoles are often indicators for autophagy [19] and contribute to the recycling of misfolded proteins and harmful cell products [20]. Interestingly, this mechanism seems to be induced by compounds **23** and **27**. The clear induction of the vacuoles by 24 h is followed by death. Light microscopic images (Figure 1) reveal multiple large vacuoles inside the schistosomula.

**Figure 2:**
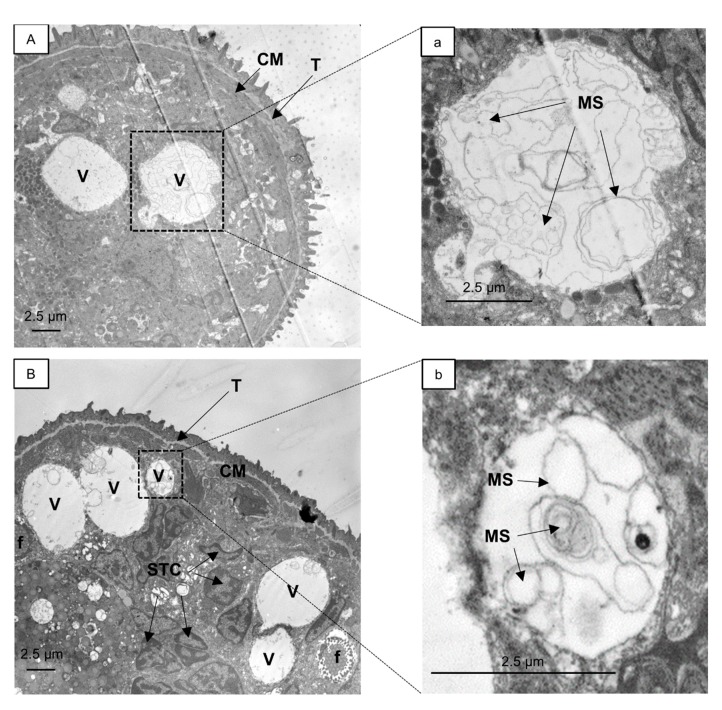
Transmission electron micrographs of *S. mansoni* schistosomula exposed for 24 h to 5 µM **27** (**A**) or **23** (**B**). Enlargements of marked areas in (**A**,**B**) are depicted in (**a**,**b**), respectively. A fusion event between vacuoles is pictured in (**B**). Vacuoles with internal membranes (**a**,**b**) and onion-like multi-lamellar structures are visible (**b**). CM, circular muscle; f, flame cell; MS, multi-lamellar structures; STC, nucleus of subtegumental cell; T, tegument; V, vacuoles.

A similar phenotype was induced by **23** (data not shown). We further analyzed the vacuoles induced by the eugenyl cinnamate **27** and thymyl cinnamate **23** using transmission electron microscopy (TEM, Figure 2). The images generated were compared to those previously reported [21]. The formation of large vacuoles in the parasite was promoted by both compounds in a similar fashion. Inside the vacuoles, resembling possible autophagolysosomes, multiple internal membranes (a,b) and onion-like multi-lamellar structures (b) were found. A fusion of two vacuoles is depicted in (B).

These observations are consistent with the description of autophagy as a defense mechanism in *Mycobacterium tuberculosis* infected macrophages by Gutierrez *et al.* [22]. Autophagy is employed as a clean-up process to remove intracellular pathogens. The characteristics of autophagosomes include a double membrane (which is digested in autophagolysosomes during autophagic flux) and the presence of cytosolic content, e.g., membranes from captured organelles [23]. Therefore, the observed vacuoles in schistosomula may represent the late stage of autophagolysosomes, which occurs after the fusion of autophagosomes with lysosomes that contain multiple hydrolases (e.g., cysteine proteases) to degrade the enveloped cytosolic material. A downstream blockade of the autophagic flux, e.g., by inhibition of the hydrolases needed to degrade the vacuole content [24], may lead to the formation of the huge vacuoles observed in schistosomula after exposure to **23** and **27**. There are few reports on autophagy in *S. mansoni*. Autophagy is inducible in *S. mansoni* schistosomula upon starvation [25] and large acidophilic compartments [26,27] are detectable using the fluorescent dye monodansylcadaverine, a relatively specific dye for autophagosomes [28]. Exposure of *S. mansoni* adult worms to astiban, lucanthone, hycanthone, and niridazole generate autophagic activities in the gastrodermis (gut) of *S. mansoni* and induce autophagic vacuoles [29]. There appear to be no reports on autophagy in schistosomes that is induced by cinnamic acid derivatives or terpenoids. Eugenol is considered to act as an antifungal [30] and antibacterial [31] as has also been suggested for thymol and other terpenoids [32], but the mechanisms of their respective actions have not been investigated. Interestingly, eugenol alone is also known to inhibit autophagy by preventing the dissociation of the Beclin1-Bcl2 heterodimer in A549 cells cotransfected with pMN-Bcl2 and pMC-Beclin1 [33]. As the Beclin1-Bcl2 complex is also present in *Schistosom*a [34] the eugenyl as well as the thymyl derivatives may influence the autophagy process in the same way. However, further studies are required.

## 3. Experimental Section

### 3.1. General Information

IR spectra were recorded on a JASCO FT/IR-6100 Fourier Transformation Infrared Spectrometer (Easton, MD, USA) equipped with an ATR unit. ^1^H (400.132 MHz) and ^13^C (100.613 MHz) NMR spectra were recorded on a Bruker Avance 400 Ultra Shield™ (Bruker Biospin, Ettlingen, Germany) spectrometer. The signals of the deuterated solvents were used as internal standards (CDCl_3_: ^1^H 7.26 ppm, ^13^C 77.0 ppm). Starting materials and reagents for the synthesis were purchased from Sigma-Aldrich (Steinheim, Germany) and VWR (Darmstadt, Germany). For TEM an EM 900 transmission electron microscope (Carl Zeiss AG, Oberkochen, Germany) was used.

### 3.2. Synthesis and Cytotoxicity Assays

The synthesis and full spectroscopic data of compounds **1**–**17**, **19**–**27** and **30** were reported previously [15]. Compounds **18**, **28** and **29** were synthesized via Steglich esterification as explained there. The protocol for the cytotoxicity studies against J774.1 was as described [35].

*Thujanyl cinnamate* (**18**): colorless sirup; IR (ATR) ν_max_ 2955, 2930, 2869, 1702, 1636, 1163 cm^−1^; ^1^H-NMR (CDCl_3_, 400 MHz) δ (ppm) 7.60 (1H, d, *J* = 16.0 Hz), 7.50 (2H, m), 7.37 (3H, m), 6.38 (1H, d, *J* = 16.0 Hz), 2.10 (1H, m), 1.87 (1H, m), 1.79 (1H, m), 1.64 (3H, s), 1.60 (1H, m), 1.39 (1H, m), 1.27 (1H, m), 0.92 (3H, d, *J* = 6.8 Hz), 0.91 (3H, d, *J* = 6.8 Hz), 0.45 (1H, m), 0.32 (1H, dd, *J* = 3.7, 5.2 Hz); ^13^C-NMR (CDCl_3_, 100 MHz) δ (ppm): 166.5, 143.5, 134.7, 130.0, 128.8 (2C), 128.0 (2C), 120.2, 91.9, 34.8, 34.6, 32.2, 31.2, 25.6, 21.5, 20.0, 19.7, 13.3.

*Thymyl phenylpropanoate* (**28**): colorless liquid; IR (ATR) ν_max_ 3027, 2962, 2925, 2869, 1754, 1505, 1497, 1413, 1148 cm^−1^; ^1^H-NMR (CDCl_3_, 400 MHz) δ (ppm) 7.35–7.24 (5H, m), 7.17 (1H, d, *J* = 7.9 Hz), 7.01 (1H, d, *J* = 7.9 Hz), 6.72 (1H, s), 3.10 (2H, m), 2.90 (2H, m), 2.80 (1H, sept, *J* = 6.9 Hz), 2.30 (3H, s), 1.12 (6H, d, *J* = 6.9 Hz); ^13^C-NMR (CDCl_3_, 100 MHz) δ (ppm): 171.6, 147.8, 140.2, 137.0, 136.5, 128.6 (2C), 128.4 (2C), 127.1, 126.4 (2C), 122.6, 35.9, 31.0, 27.0, 23.0 (2C), 20.8.

*Eugenyl phenylpropanoate* (**29**): colorless liquid; IR (ATR) ν_max_ 2963, 2936, 2924, 1756, 1503, 1122 cm^−1^; ^1^H-NMR (CDCl_3_, 400 MHz) δ (ppm) 7.36-7.23 (5H, m), 6.90 (1H, d, *J* = 8.0 Hz), 6.80 (1H, d, *J* = 1.9 Hz), 6.78 (1H, dd, *J* = 1.9, 8.0 Hz), 5.98 (1H, m), 5.14 (1H, m), 5.10 (1H, m), 3.80 (3H, s), 3.40 (2H, m), 3.12 (2H, m), 2.93 (2H, m); ^13^C-NMR (CDCl_3_, 100 MHz) δ (ppm): 171.1, 150.9, 140.4, 139.0, 138.0, 137.1, 128.5 (2C), 128.4 (2C), 126.3, 122.5, 120.7, 116.1, 112.7, 55.8, 40.1, 35.6, 31.0.

### 3.3. Schistosoma Screens and TEM

Maintenance of the *S. mansoni* life cycle, preparation of schistosomula and adult worms, compound storage and treatment of schistosomula and adult worms were as described [16]. Compounds were dissolved in DMSO. Control assays contained the same amount of DMSO. The compound names, structures and phenotypes arising are listed in Table 1 and Table 2. PZQ served as anti-schistosomal reference drug. TEM studies were performed as follows. After treatment with compound **27** for 24 h schistosomula were fixed for 45 min with 2.5% glutaraldehyde-50 mM cacodylate (pH 7.2; Sigma-Aldrich, Steinheim, Germany) at room temperature and then contrasted for 2 h at 4 °C with 2% OsO_4_ buffered with 50 mM cacodylate (pH 7.2). The material was washed with distilled water, and incubated overnight at 4 °C with 0.5% uranyl acetate in distilled water. The parasites were dehydrated and embedded in EPON. Ultrathin sections were mounted on 300-mesh grids, stained with uranyl acetate and lead citrate, and analyzed with an EM 900 transmission electron microscope (Carl Zeiss AG, Oberkochen, Germany).

## 4. Conclusions

We screened a library of 30 derivatives of bornyl caffeate (**1**) for anti-schistosomal activity against schistosomula and adult worms of *S. mansoni*. Adults were refractory to all of the compounds. For schistosomula, the compound eugenyl cinnamate (**27**) was lethal. Prior to death, this compound and thymyl cinnamate (**23**) induced the appearance of multiple internal vacuoles, which may indicate an induction of autophagy, although this hypothesis requires formal testing. No direct correlation between cytotoxicity (*vs.* J774.1 cells) and anti-schistosomal activity was found. Further studies with an expanded series of compounds and including dose-response relationships are necessary to determine whether these compounds represent a basis for future anti-schistosomal drugs.

*Sample Availability*: Samples of all synthesized compounds are available from the authors.
